# Combined Effects of Irrigation Regime, Genotype, and Harvest Stage Determine Tomato Fruit Quality and Aptitude for Processing into Puree

**DOI:** 10.3389/fpls.2017.01725

**Published:** 2017-10-05

**Authors:** Alexandre Arbex de Castro Vilas Boas, David Page, Robert Giovinazzo, Nadia Bertin, Anne-Laure Fanciullino

**Affiliations:** ^1^UR 1115 Plantes et Systèmes de cultures Horticoles, Institut National de la Recherche Agronomique, Centre PACA, Avignon, France; ^2^UMR 408 Sécurité et Qualité des Produits d'Origine Végétale, INRA, Centre PACA, Université d'Avignon, Avignon, France; ^3^Société Nationale Interprofessionnelle de la Tomate, Avignon, France

**Keywords:** quality, *Solanum lycopersicum*, deficit irrigation, pre- and post-harvest links, antioxidants, thermal processing, consistency

## Abstract

Industry tomatoes are produced under a range of climatic conditions and practices which significantly impact on main quality traits of harvested fruits. However, the quality of tomato intended for processing is currently addressed on delivery through color and Brix only, whereas other traits are overlooked. Very few works provided an integrated view of the management of tomato puree quality throughout the chain. To gain insights into pre- and post-harvest interactions, four genotypes, two water regimes, three maturity stages, and two processes were investigated. Field and glasshouse experiments were conducted near Avignon, France, from May to August 2016. Two irrigation regimes were applied: control plants were irrigated in order to match 100% of evapotranspiration (ETP); water deficit (WD) plants were irrigated as control plants until anthesis of the first flowers, then irrigation was reduced to 60 and 50% ETP in field, and glasshouse respectively. Fruits were collected at three stages during ripening. Their color, fresh weight, dry matter content, and metabolite contents were determined before processing. Pericarp cell size was evaluated in glasshouse only. Two laboratory-scaled processing methods were applied before structural and biochemical analyses of the purees. Results outlined interactive effects between crop and process management. WD hardly reduced yield, but increased dry matter content in the field, in contrast to the glasshouse. The puree viscosity strongly depended on the genotype and the maturity stage, but it was disconnected from fruit dry matter content or Brix. The process impact on puree viscosity strongly depended on water supply during fruit production. Moreover, the lycopene content of fresh fruit may influence puree viscosity. This work opens new perspectives for managing puree quality in the field showing that it was possible to reduce water supply without affecting yield and to improve puree quality.

## Introduction

There is much interest in improving fruit and vegetable quality through sustainable means in order to meet future food needs and tackle environmental challenges. While about 800 million people are undernourished globally (Welch and Graham, 1999; McGuire, 2015), up to one third of food is never consumed (FAO, 2011), especially fruits and vegetables, which are naturally rich in major phytonutrients. Food losses occur throughout the supply chain: during harvest, during post-harvest handling and storage, during processing and at distribution and consumer levels. Therefore, processing fruits and vegetables represent a strategic approach to meet nutritional needs of the growing population, considering their availability all along the year, and provided that organoleptic and nutritional properties are preserved during processes. Processing tomato is a major crop that represents the principal source of important phytonutrients such as β-carotene and lycopene (Dorais et al., 2008). The most part is consumed as tomato puree, paste, or sauce (Mirondo and Barringer, 2015).

In addition, a major environmental concern in agriculture is the use of fresh water for irrigation (Postel et al., 1996). Water resources are under threat due to the increase in water demand for agriculture, and the gap between water availability and demand is exacerbated by global climate changes (Afzal et al., 2016). Processing tomato, an intensive production in terms of water use, is highly concerned with this issue (Rinaldi et al., 2007). For example, in Italy, the blue water footprint (ratio of the volume of irrigation to the crop yield) of this production has been estimated at 60 m^3^ per ton (Aldaya and Hoekstra, 2010). In line with this, considerable efforts have been made for increasing water use efficiency of tomato crop (Stikic et al., 2003; Costa et al., 2007; Rinaldi et al., 2007; Patanè and Cosentino, 2010; Patanè et al., 2016). These works underlined that water deficit (WD) is one of the main limiting factors affecting the yield of processing tomato (Costa et al., 2007; Patanè and Cosentino, 2010). The yield reduction depends on water deficit intensity and duration as well as on its timing during tomato development (Rinaldi et al., 2007; Patanè and Cosentino, 2010). WD reduces the weight of individual fruits more than the number of fruits per plant (Casa and Rouphael, 2014). Because mild WD decreases fruit water accumulation more than dry mass accumulation, the decrease in yield may in fact turn out positive for processing. Indeed, tomato process includes a phase of dehydration/concentration, and reducing water content of raw material makes the process more efficient. In addition, many other traits related to fruit quality (soluble solid content and titratable acidity) and to fruit nutritional value (vitamin C and carotenoids) are affected by WD depending on climatic conditions, cultivars, or fruit developmental stages (Garcia and Barrett, 2006; Patanè and Cosentino, 2010; Anthon et al., 2011; Patanè et al., 2011; Barbagallo et al., 2013). Several works have analyzed the effect of one specific pre-harvest factor on quality traits of both fresh fruits and processed purees. According to Patanè and Cosentino (2010) WD decreased Bostwick consistency of purees. Large variations in paste quality traits (color, consistency, soluble solid content, pH and titratable acidity) were found among cultivars and among maturity stages (Garcia and Barrett, 2006). Delayed harvesting caused a rise in pH and a loss of citric acid (Anthon et al., 2011). Therefore, considering production management as a levy to monitor the quality of processed fruits may lead to innovative strategies to improve puree quality.

High viscosity, fresh flavor and retention of natural color are important quality traits of ketchup and tomato puree (Chong et al., 2009). Interestingly, color shift during processes results from putative modifications of lycopene storage structures (more easily extractable) rather than from variations in lycopene content (Svelander et al., 2010; Page et al., 2012; Makroo et al., 2017). Concerning viscosity, dehydration during processing has a major influence. Relationships between dry matter content and viscosity on one hand, and between dry matter content and soluble solid content (SSC, in °Brix) on the other hand, are well-known from manufacturers since puree price is based on °Brix. However, SSC is not the only factor affecting rheology (Barrett et al., 1998). Processing parameters such as breaking temperature and dynamic sieving modify the water soluble/insoluble solid content ratio, particle sizes and pectin state, which, in turn, affect the puree rheology (Sanchez et al., 2002; Moelants et al., 2014). Those physicochemical variables depend on the biological structures of fruit tissues and their reactivity to the process. For example, breaking temperature is currently used to modulate the consistency of tomato products: a high temperature treatment, immediately after fruit crushing (hot break, HB: 90°C) produces much more viscous purees than cold break (CB) treatment, where fruits are first crushed and then macerated at moderate temperature (70°C; Moelants et al., 2014).

The quality of tomato puree is built throughout the food chain. Yet, very few works have simultaneously analyzed factors affecting fruit quality during the growing season and those that operate during processing. Currently, in industry, the quality of processed fruits is assessed through the color and Brix index only, whereas other physical, structural and biochemical traits are overlooked. In order to better understand variations in puree quality, insights into pre- and post-harvest interactions should be gained. To fulfill this objective, we investigated major traits of fruit quality in response to water supply, genotypes and ripening stages, and we assessed their impact on puree quality obtained from HB and CB processes. The response to WD under different climatic conditions was assessed by conducting both field and glasshouse experiments.

## Materials and methods

Two experiments were conducted concurrently in spring and summer 2016, the first in the field according to commercial practices and the second in a glasshouse under controlled climatic conditions. In both experiments, four industry-type (determinate) cultivars of *Solanum lycopersicum*, namely “H1015,” “H1311,” “Miceno,” and “Terradou,” were selected on the basis of a previous study (not published), based on the contrasted purees obtained: from low (“Terradou”) to medium (“H1015” and “Miceno”), or high (“H1311”) lycopene content and from low (“Terradou”) to medium (“H1015” and “Miceno”) or high viscosity (“H1311”). Those four genotypes were studied under two levels of irrigation, control and water deficit (WD). All seeds were germinated under standard glasshouse conditions (25°C day, 15°C night) near Avignon, France, in April 2016.

### Open-field experiment

**Figure 1:**
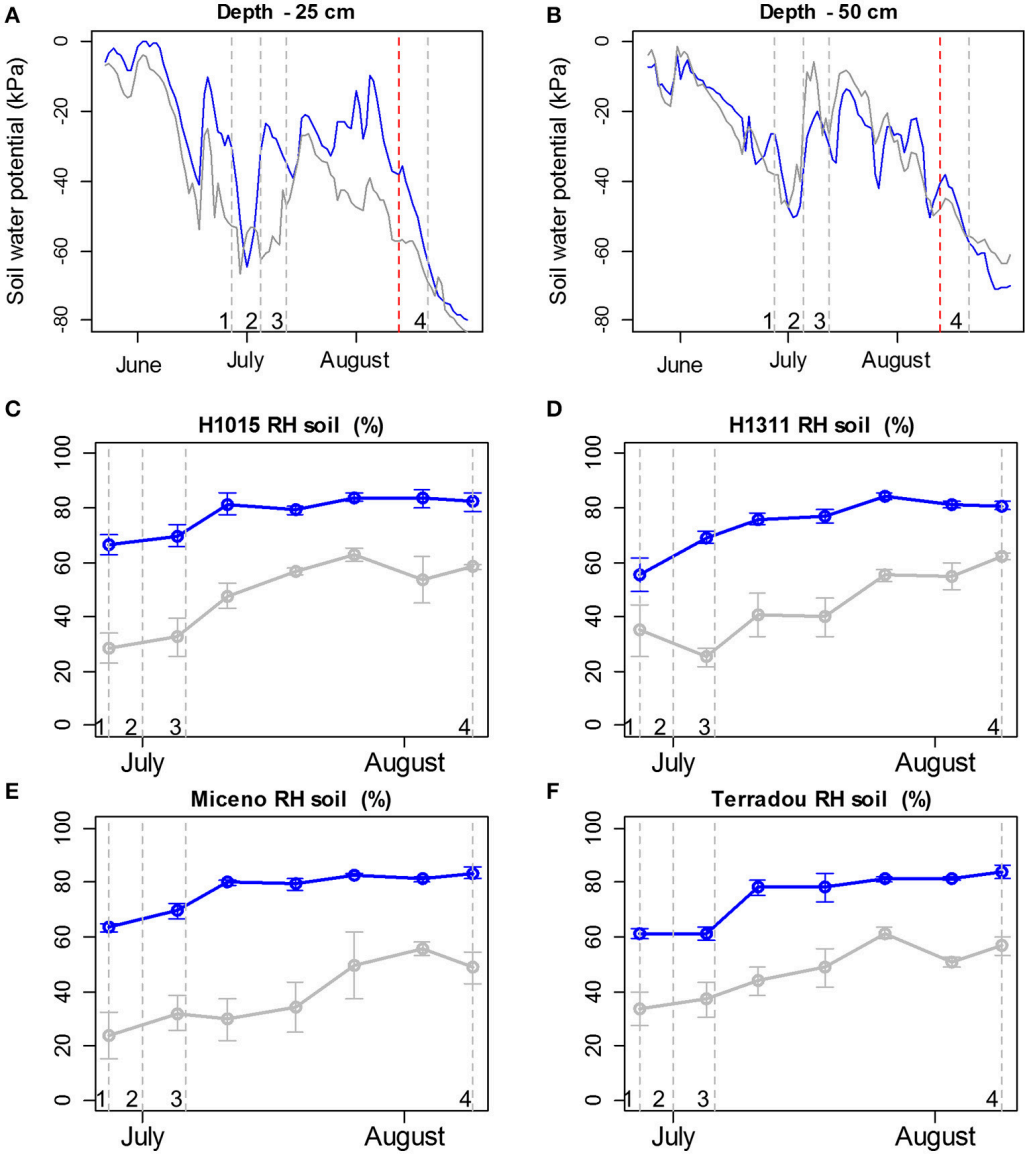
Variations in soil humidity under field **(A,B)** and glasshouse **(C–F)** conditions. **(A,B)** Soil water potentials determined with 6 Watermark sensors installed at 25 **(A)** and 50 **(B)** cm depths under the well-watered regime (blue lines) and the water deficit regime (gray lines). **(C–F)** soil relative humidity (RH) determined weekly in 9 pots for the 4 cultivars (“H1015,” “H1311,” “Miceno,” and “Terradou”) and the 2 water regimes: well-watered (blue lines) and water deficit (gray lines). Note that a RH of 80% corresponds to the maximum of soil water retention. Mean values are reported ± SE. On **(A–F)**, vertical gray lines with number 1, 2, 3, and 4 indicate anthesis of the first, second, third truss, and the harvest of those trusses respectively. The vertical red line on **(A,B)** indicates the end of irrigation.

Two blocks of 1,800 plants each (450 plants per cultivar) were designed in an experimental field near Avignon (43°54′N 4°52′E), France. Each block was 7 m wide and 90 m long and surrounded by border plants. The four genotypes and the two irrigation regimes were randomly distributed within the two blocks. All plants were grown under identical field conditions: 900 plants per genotype were transplanted in May 2016 at a density of 3.3 plants m^−2^, fertilizers (86 kg.ha^−1^ of N, 33 kg.ha^−1^ of P, and 198 kg.ha^−1^ of K) were supplied before transplanting and insects and diseases were controlled according to current practices. The water irrigation was supplied by a drip irrigation system. Irrigation was scheduled daily to compensate the evapotranspiration loss from tomato crop (ETP). ETP was determined daily using reference evapotranspiration estimated from the Penman–Monteith equation (Monteith, 1965) and taking into account crop coefficient (Kc) and precipitations. The variations of Kc during the season are given in Supplementary Figure 1. Daily variations in air temperature, rainfall and solar radiation are reported in Supplementary Figure 2. Water was first supplied every day in order to fully fit 100% of ETP. Forty-five days after sowing, two levels of irrigation were applied: (1) water deficit (60% replacement of ETP) and (2) well-watered to match 100% replacement of ETP (Supplementary Figure 3). To mimic current production practices, irrigation was stopped 1 week before harvest. The soil water potential was recorded hourly using Watermark (Campbell Scientific, Antony, France) soil moisture sensors (six per water regime), which were installed at 25 and 50 cm depths. The records showed reduced soil humidity at 25 cm depth when compared to the control (Figure 1). Control plants experienced higher soil water potentials than plants under WD at 25 cm depth (Figure 1A). Under well-watered condition, 50% of the data (between the upper and the lower quartile) ranged from −38 to −13 kPa, while under WD 50% of the data ranged from −57 to −27 kPa. At 50 cm depth, soil water potentials remained similar between the two irrigation treatments (Figure 1B).

Three independent samples of 15 fruits from each treatment, genotype, and block were harvested at 40 (light orange), 47 (orange red), and 55 (red ripe) days after anthesis (DAA) for quality analyses and processing.

### Glasshouse experiment

Eighteen plants per genotype were grown in 4 L pots filled with compost (substrate 460, Klasmann, Champety, France) at a density of 1.8 plant.m^−2^ under glasshouse conditions near Avignon (43°54′N 4°52′E), France. Day-night temperature controls were set at 25–15°C and the air humidity ranged between 30 and 95%. Solar radiation inside the glasshouse varied from 7.3 to 26.2 MJ m^−2^ day^−1^ (Supplementary Figure 4). Flowers were pollinated three times a week using an electrical bee. Plants were supplied daily with a nutrient solution (Liquoplant Rose, Plantin, Courthézon, France). This solution was diluted between 4‰ (${\text{NO}}_{3}^{-}$, 1.7 mM; ${\text{NH}}_{4}^{+}$, 1.3 mM; P_2_O_5_, 0.5 mM; K_2_O, 2.2 mM; MgO, 0.9 mM, and FeEDTA, 15 μM) and 8‰ (${\text{NO}}_{3}^{-}$, 3.4 mM; ${\text{NH}}_{4}^{+}$, 2.6 mM; P_2_O_5_, 1 mM; K_2_O, 4.4 mM; MgO, 1.8 mM, and FeEDTA, 30 μM) according to the plant developmental stage, which corresponded to an average electro conductivity of 1.8 dS m^−1^. First, all plants were irrigated in order to match 100% replacement of ETP. ETP was determined daily using reference evapotranspiration estimated from the Penman–Monteith equation (Monteith, 1965) and taking into account Kc. Soil relative humidity and drainage were maintained around 70% (maximum water retention capacity of the substrate) and 15%, respectively. After 30 days (corresponding to the anthesis of the first flowers), a water deficit treatment was applied to half of the plants. The irrigation was reduced to 50% of ETP for plants under WD, while maintained at 100% of ETP for control plants from 30 to 120 days after planting (until end of fruit ripening; Supplementary Figure 3). The drip irrigation system was scheduled to irrigate every 0.25 mm. Soil relative humidity was measured weekly between 9 and 10 a.m. (solar time) in all pots using water content sensors (WCM-control, Grodan, Roermond, The Netherlands). Figures 1C–F shows changes in soil relative humidity during the treatment. A 25–60% reduction in soil relative humidity was monitored in all pots following WD treatment (gray lines). The electro-conductivity of both limited-water and well-watered pots ranged between 1 and 2 dS m^−1^ until 90 days after planting and between 2 and 3 thereafter.

For fruit quality analyses and processing, four independent samples of 15 fruits were harvested from 9 plants at three stages: 40, 47, and 55 DAA.

### Plant and fresh fruit physiological measurements

During the WD treatment, from June to August 2016, leaf and stem water potentials, leaf conductance and fruit growth were monitored on control and WD plants. Measurements of leaf conductance were conducted between 9 and 10 a.m. (solar time) using an AP4 porometer (Delta-T Devices Ltd, Cambridge, England), while measurements of water potentials were performed between 12 and 13 p.m. (solar time) using a pressure chamber (Scholander et al., 1965). Every week, 12 newly mature leaves from six individuals were marked for each treatment, genotype, and block. Half of the marked leaves were used for leaf conductance (g_s_) and midday leaf water potential (ψ_Lmidday_) measurements, while six other leaves were covered with aluminum foil and plastic bags to allow leaf and stem water potentials to equilibrate at least 2 h before stem water potential measurements (ψ_Smidday_). At least three mature leaves from three individuals per treatment, per genotype, and per block were sampled at the end of the experiment for specific leaf area (SLA) determination.

Fruit growth was measured weekly with a digital caliper between 9 and 10 a.m. (solar time). Three fruits from three different trusses were monitored on three plants per treatment, per genotype, and per block. Flowers were labeled at anthesis at three different dates, so that fruits at three stages around maturity (light orange, orange red, and red ripe) could be collected on the same day in August. Consequently, all harvested fruits underwent similar environmental conditions from anthesis to harvest (Figure 1).

At the end of the experiment in August, at least four plants per treatment, per genotype, and per block were collected for fresh and dry yield determination.

### Fresh fruit quality

Fruit color, dry matter, starch, soluble sugar, organic acid, and carotenoid contents were analyzed in both experiments. The color was measured with a Minolta CR.400 calibrated with a standard background. At least three fruits from the three or four samples were tested three times at equidistant points along the equatorial plane. The dry matter content was determined by weighting 3 g of fruit pericarp pieces before and after drying at 85°C. For biochemical analyses, pieces of fruit pericarp were immediately frozen and kept at −80°C. Soluble sugars, starch and organic acids were extracted according to the method described by Gomez et al. (2002) and analyzed by HPLC (Waters 410, Part WAT070390, Milford, U.S.A.). Carotenoids were extracted according to the method described by Sérino et al. (2009) modified by Page et al. (2012) for quantification against internal standard (apo_8′carotenal) after HPLC separation on a C30-column (Develosil® C30-UG-3, Nomura chemical CO., Seto, Japan) with a UV-visible detector (SPD-M20A; Shimadzu, Kyoto, Japan). The pericarp cell number was measured after tissue dissociation according to a method adapted from Bünger-Kibler and Bangerth (1983). Cells were counted using a microscope equipped with a camera (QImaging, Surrey, Canada) and Qcapture Pro 6.0 software (QImaging, Surrey, Canada; Bertin et al., 2002).

### Tomato processing and quality

Purees were prepared by either hot break (HB) or cold break (CB) treatment according to a laboratory scaled method described by Page et al. (2012). Fruits were cut into large pieces (around 2 cm^3^), mixed altogether, and split into two identical batches of 400 g each. For HB, one batch was first heated until boiling temperature in a microwave oven (900 w, full power, 0.9 s g^−1^ of tomato), then chopped for 30 s in a Waring® blender. For CB, the other batch was first chopped at room temperature for 30 s in the same Waring® blender and then heated for the same duration and conditions than for HB. Both purees were then passed through a hand-held potato masher with a 2 mm grid to remove skins and seeds, stored into a 500 ml glass jar with sealed lid, sterilized for 15 min at 100°C in a laboratory scaled autoclave, and stored at 4°C before analysis. The grinding step at room temperature in CB process allowed for the reaction of fruit intrinsic enzymes (especially polygalacturonase and pectin-methyl esterase) on cell walls, and therefore leads to lower consistency of purees compared to HB ones (Anthon et al., 2002). Consequently, the enzymatic potential of fruits was indirectly estimated as the difference in puree consistency between HB and CB processes, in our standardized conditions. The color of the purees was measured with a Minolta CR.400 using a specific cuvette for measurement of liquid or paste color and calibrated against a white background. Color results were expressed in the CIE L ^*^ a ^*^ b ^*^ color space. Color coordinates were used to calculate the hue angle (H°), which identifies the color at a 360° angle (McGuire, 1992). The dry matter content was determined by weighting around 3 g of fruit puree before and after drying for 3 days at 85°C. The soluble solid content (SSC) was measured by refractometry with an ATAGO PR-1000 digital refractometer with automatic temperature compensation at 25°C and results were expressed in degree Brix, according to AOAC (2002). Rheological behavior of the puree was assessed through two characteristic measurements: (1) consistency was measured using a Bostwick consistometer (CSC Scientific Company, Fairfaix, USA) and according to manufacturer's manual, results were expressed as arbitrary Bostwick unit (Bw). The lower the Bostwick value, the higher the puree consistency; (2) the viscosity was calculated from a steady state measurement performed on an Anton Paar MCR 301 viscosimeter (Graz, Austria), with a double ribbon impeller (with an inner radius of 11 mm, a pitch of 45 mm, a length of 45 mm, and an outer stationary cup with an outer radius of 14.46 mm). A flow curve was registered between 0.1 and 100 s-1, 50 points and 5 s per point. Flow properties were described by the Herschel–Bulkley model (Espinosa et al., 2011).

### Data analysis

Data were analyzed using R statistical software (http://www.R-project.org). Physiological traits, data of yield, and quality traits were analyzed by analysis of variance (the agricolae R package and aov function; De Mendiburu, 2014). Heteroscedasticity and normality tests were performed before model evaluation. Regarding field experiment, when the ANOVA F-test showed no significant difference in means between the blocks, data from block 1 and 2 were pooled. Tables 1, 2 synthesize results from univariate ANOVAs. Multiple comparison of means was performed using the Least Significant Differences (LSD) test (α = 0.05). When heteroscedasticity was detected, we used the Kruskal–Wallis non-parametric test followed by multiple comparisons of means through a t-student test on the ranks (α = 0.05).

**Table 1:** Results of univariate analyses of variance (ANOVAs) for the plant traits measured under field and glasshouse conditions.

**Factors**	**WD**		**Genotype**		**Interaction (WD** × **Genotype)**		**R^2^ (%)**
	***p*-value**	**SSx/SStotal%**	***p*-value**	**SSx/SStotal%**	***p*-value**	**SSx/SStotal%**	
**FIELD**							
Stomatal conductance g_s_	<2e-16^***^	55.65	0.14	2.68	0.96	0.14	58.47
Individual leaf dry weight	6.82e-10^***^	50.79	0.00371^**^	12.27	0.04935^*^	6.62	69.68
Individual leaf area	<2e-16^***^	76.61	0.26302	1.69	0.40959	1.20	79.51
Specific leaf area	0.3428	1.62	0.0342^*^	16.75	0.1278	10.59	28.96
Total fruit biomass (g FW per plant)	0.865	0.03	5.19e-06^***^	38.03	0.608	2.01	40.07
Total fruit biomass (g DW per plant)	0.000315^***^	12.02	6.1e-08^***^	41.52	0.583241	1.60	55.14
Total fruits (number per plant)	0.298	1.15	1.41e-06^***^	40.97	0.987	0.14	42.27
Individual fruit fresh weight	0.174	1.88	5.15e-07^***^	42.75	0.368	3.20	47.83
**GLASSHOUSE**							
Leaf water potential ψ_L_	<2e-16^***^	52.72	5.95e-07^***^	4.63	0.352	0.46	57.81
Stem water potential ψ_S_	<2e-16^***^	28.79	0.00351^**^	3.08	0.39672	0.66	32.53
Stomatal conductance g_s_	<2e-16^***^	33.88	0.0453^*^	1.72	0.822	0.19	35.79
Total plant biomass (g FW per plant)	0.000543^***^	16.27	0.371852	3.91	0.784159	1.31	21.49
Total plant biomass (g DW per plant)	0.441	0.83	0.126	8.19	0.573	2.77	11.79
Total fruit biomass (g FW per plant)	1.23e-10^***^	44.74	0.135	4.39	0.415	2.20	51.33
Total fruit biomass (g DW per plant)	3.2e-09^***^	38.01	0.0255^*^	8.01	0.4095	2.36	48.39
Total fruits (number per plant)	1.02e-06^***^	27.85	0.0447^*^	8.12	0.371	3.04	39.01
Individual fruit fresh weight	1.95e-09^***^	33.14	5.91e-06^***^	22.58	0.743	0.85	56.57

*R^2^ (%) Proportion of total variance explained by the model. SS, Sum of squares; SSx/SStotal %, the proportion of the explained variance*.

Significance codes: 0 “***” 0.001 “**” 0.01 “*”.

**Table 2:** Results of univariate analyses of variance (ANOVAs) for the quality traits measured under field and glasshouse conditions.

**Factors**	**WD**		**Genotype**		**Stage**		**Interaction (WD** × **Genotype)**		**Interaction (WD** × **Stage)**		**Interaction (Genotype** × **Stage)**		**Interaction (WD** × **GenotypexStage)**		***R^2^***
	***p*****-value**	**SSx**/**SStotal %**	***p*****-value**	**SSx**/**SStotal %**	***p*****-value**	**SSx**/**SStotal %**	***p*****-value**	**SSx**/**SStotal %**	***p*****-value**	**SSx**/**SStotal %**	***p*****-value**	**SSx**/**SStotal %**	***p*****-value**	**SSx**/**SStotal %**	
**FIELD**															
Brix Fresh Fruit	0.45581	0.15	0.45581	2.35	1.594e-07^***^	56.13	0.64208	1.48	0.57835	0.98	0.04545^*^	13.45	0.52889	4.56	79.10
Starch	0.1858229	1.50	0.0001169^***^	26.03	0.0003225^***^	18.55	0.2854716	3.25	0.5404158	1.02	0.0018167^**^	24.45	0.3486161	5.75	80.56
Glucose	0.612931	0.30	0.000515^***^	28.86	0.021832^*^	10.23	0.49786	2.78	0.256552	3.27	0.051573^.^	16.94	0.213701	10.37	72.75
Fructose	0.7798865	0.09	0.0004726^***^	29.67	0.0136013^*^	11.89	0.5626139	2.41	0.3868617	2.27	0.0687178^.^	15.81	0.2269084	10.22	72.38
Citric	0.6517	0.42	0.1284	12.59	0.3449	4.47	0.8127	1.91	0.8508	0.65	0.2421	17.32	0.3436	14.38	51.76
Malic	0.56157	0.73	0.03647^*^	21.10	0.21523	6.92	0.74182	2.65	0.9773	0.10	0.4821	11.98	0.8321	5.81	49.29
Hue Fresh Fruit	0.9673	0.00	0.7357	0.37	1.909e-14^***^	89.00	0.643	0.49	0.912	0.05	0.539	1.48	0.451	1.72	93.10
Lycopene	0.2719442	0.85	4.95e-08^***^	56.71	0.0009003^***^	12.79	0.9728094	0.15	0.9099104	0.13	0.0406639^*^	10.68	0.6972749	2.58	83.89
Brix CB	0.069902^.^	1.50	2.737e-11^***^	74.70	0.001186^**^	7.52	0.478907	1.06	0.410176	0.77	0.262013	3.45	0.864551	1.03	90.02
Hue CB	0.8297	0.02	3.839e-07^***^	33.64	5.163e-09^***^	46.87	0.5709	1.03	0.9863	0.01	0.2021	4.68	0.7433	1.74	87.99
Viscosity CB	0.0283^*^	4.60	0.0512^.^	7.55	5.739e-08^***^	61.00	0.782	0.91	0.2575	2.42	0.8803	1.96	0.9506	1.31	79.75
Brix HB	0.12391	1.23	1.926e-10^***^	71.87	0.01765^*^	4.63	0.40413	1.47	0.12056	2.23	0.30448	3.70	0.3727	3.29	88.42
Hue HB	0.6248	0.12	1.425e-06^***^	28.44	7.613e-10^***^	56.24	0.61	0.91	0.8623	0.15	0.7356	1.74	0.9766	0.56	88.17
Viscosity HB	1.266e-06^***^	5.63	8.655e-15^***^	51.50	9.928e-13^***^	29.64	0.84965	0.11	0.02576^*^	1.17	3.026e-05^***^	7.51	0.25536	1.15	96.71
**GLASSHOUSE**															
Brix Fresh Fruit	0.05954^.^	2.88	8.08e-05^***^	19.60	0.28507	2.01	0.31688	2.82	0.95269	0.08	0.56471	3.83	0.02538^*^	12.18	43.39
Starch	0.9717	0.00	0.5116	2.26	0.0183^*^	8.23	0.2386	4.20	0.2652	2.63	0.2636	7.65	0.5288	5.02	29.98
Glucose	0.14498	1.51	2.931e-07^***^	29.19	0.6562	0.59	0.52891	1.55	0.26001	1.91	0.07321^.^	8.46	0.11715	7.40	50.61
Fructose	0.06437^.^	1.87	1.246e-11^***^	42.14	0.57975	0.58	0.3772	1.66	0.16636	1.95	0.02459^*^	8.25	0.09310^.^	6.02	62.46
Citric	0.40156	0.36	1.639e-13^***^	49.78	0.23954	1.46	0.41953	1.43	0.02681^*^	3.81	0.1827	4.58	0.42107	3.05	64.47
Malic	0.421699	0.48	1.165e-05^***^	22.63	0.746217	0.44	0.228504	3.28	0.214946	2.33	0.74837	2.56	0.004073^**^	15.70	47.41
Hue Fresh Fruit	0.023877^*^	2.74	0.000181^***^	11.69	6.856e-12^***^	38.65	0.908774	0.28	0.249043	1.46	0.291466	3.86	0.237691	4.24	62.92
Lycopene	0.03946^*^	1.53	<2e-16^***^	63.96	0.33174	0.78	0.21975	1.57	0.34003	0.76	0.07845^.^	4.14	0.36543	2.31	75.03
Brix HB	3.023e-05^***^	14.06	1.278e-05^***^	21.41	0.01586^*^	6.23	0.60554	1.31	0.869	0.20	0.71807	2.61	0.62031	3.14	48.97
Hue HB	0.0311284^*^	1.70	2.761e-12^***^	30.22	3.275e-12^***^	27.51	0.0007376^***^	6.66	0.4658811	0.54	0.0430425^*^	4.87	0.1988222	3.12	74.64
Viscosity HB	0.010617^*^	2.44	3.327e-13^***^	33.90	3.258e-12^***^	27.75	0.428888	1.00	0.720723	0.23	0.001505^**^	8.62	0.966472	0.48	74.43

*R^2^ (%) Proportion of total variance explained by the model. SS, Sum of squares; SSx/SStotal %, the proportion of the explained variance*.

Significance codes: 0 “^***^” 0.001 “^**^” 0.01 “^*^” 0.05 “^.^”.

The Factomine R package and the plot PCA function were used to perform PCA analysis. Data from field were composed of 15 variables of fruit and puree quality and 48 observations (4 cultivars × 3 stages × 2 blocks × 2 irrigation levels). Data (means of 3 biological replicates) were centered and scaled by variables. Data from glasshouse are presented in Supplementary Figure 5. Eigen values and contribution of variables to each dimension are reported in Supplementary Tables 1, 2 for field and glasshouse, respectively.

Pearson correlation was performed to investigate links between fresh fruit and puree quality traits. The coorplot package was used to draw the correlation matrix of the quality traits (Wei et al., 2016). The GGMselect, GeneNet, and igraph packages (Schaefer et al., 2013; Csardi and Nepusz, 2014; Bouvier et al., 2016) were used to build a partial correlation network on fruit and puree quality traits based on the residues of the linear regressions (elimination of the genotype and treatment effects). Correlation matrix and partial correlation network were performed independently for field and glasshouse experiments.

## Results

### Yield and fruit composition were not much affected by water deficit, despite significant plant responses

Univariate ANOVAs were performed on plant traits to analyze the effects of WD, genotype and WD × genotype interactions in the field and glasshouse experiment, separately (Table 1). In field, no block effect was found. This factor was omitted in Table 1. None of the WD × genotype interactions were significant, except for individual leaf dry weight measured in field (Table 1). During the decline in soil humidity in the WD treatment, physiological traits were highly affected by WD (*p* ≤ 0.001). A 50% reduction in stomatal conductance (g_s_) was observed for all cultivars in both open-field and glasshouse experiments (not shown). Accordingly, significant reductions in individual leaf area (from −22 to −40% according to the genotype) and individual leaf dry weight (from −14 to −39%) were observed under WD treatment in field. These reductions did not result in significant changes in specific leaf area (Table 1). In glasshouse, all cultivars experienced variations in water status. Differences of midday leaf and stem water potentials (ψ_Lmidday_ and ψ_Smidday_) between control and WD plants were significant (*p* ≤ 0.001). The highest variations were registered for ψ_Lmidday_, which ranged from −0.7 to −0.5 MPa for control plants and from −1.0 to −0.6 MPa for WD plants. In the glasshouse, the total plant fresh biomass was reduced under WD treatment (up to −44% for “Miceno”).

**Figure 2:**
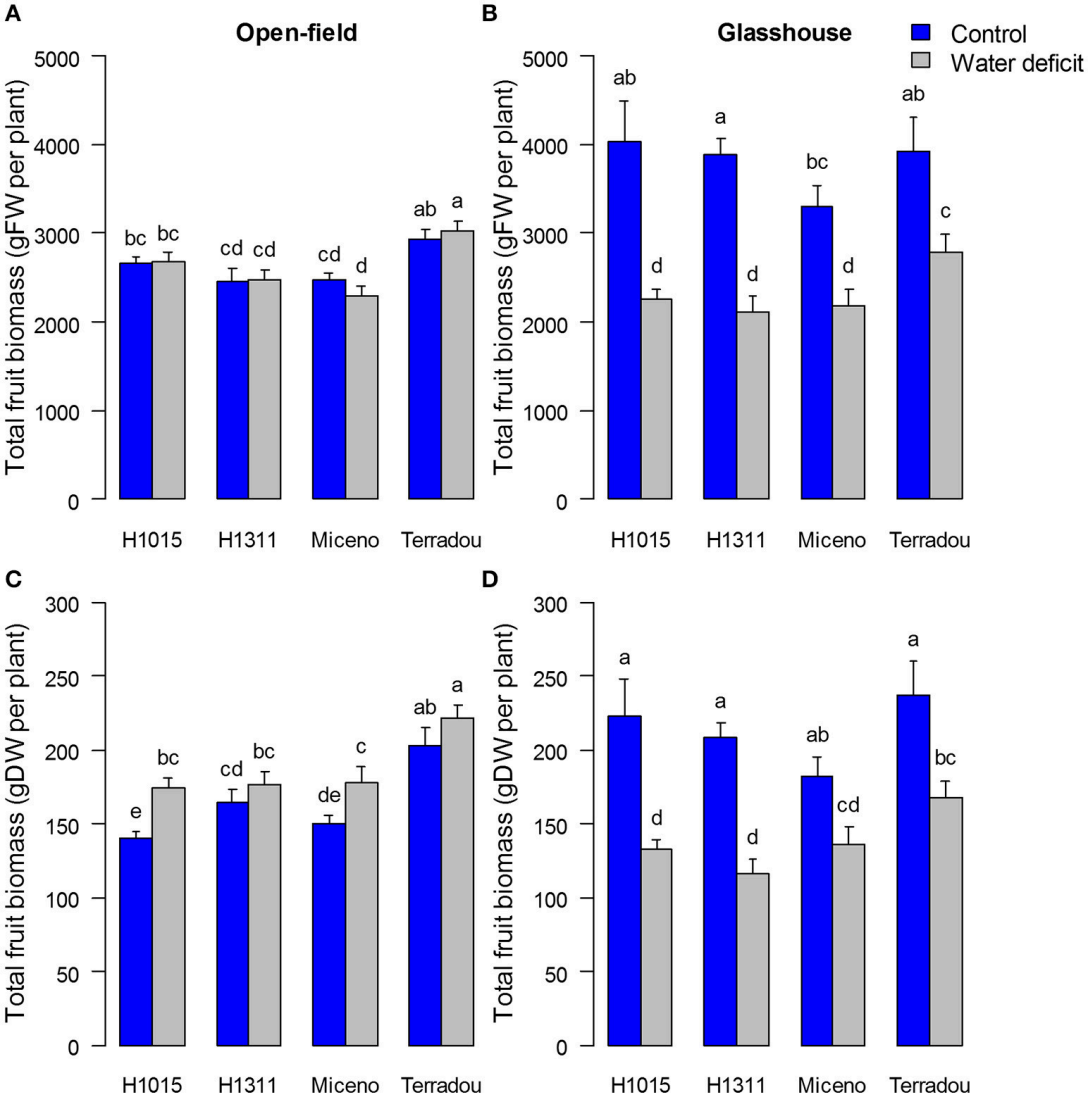
Impact of water deficit and cultivar on fresh **(A,B)** and dry **(C,D)** yield expressed as total fruit biomass (g) per plant under field **(A,C)** and glasshouse **(B,D)** conditions. Note that the color code is blue for well-watered plants and gray for plants under water deficit. Values are means of *n* ≥ 8 ± SE. Bars marked by different letters indicate significant different values (Kruskal–Wallis test, α = 0.05).

**Figure 3:**
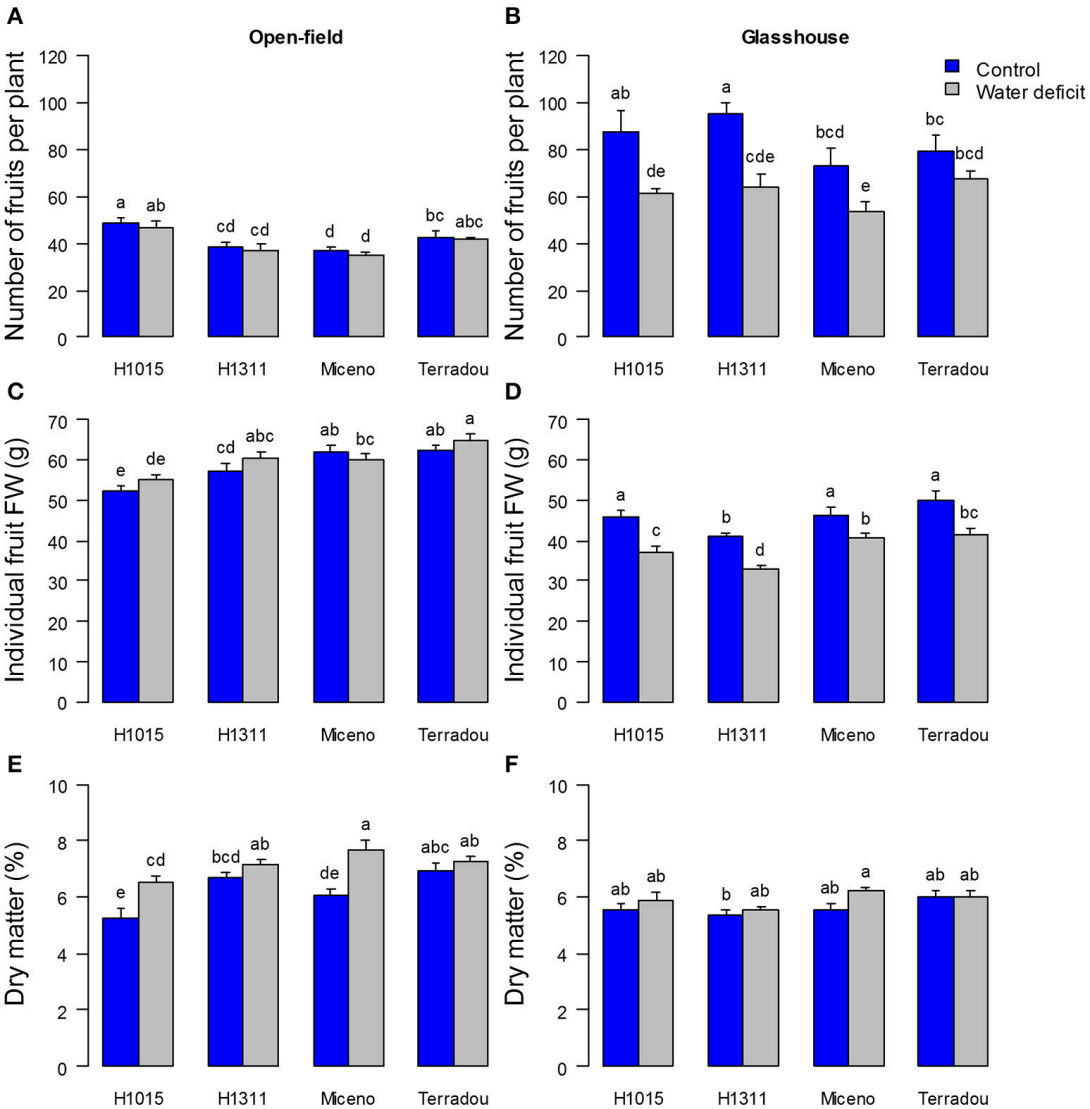
Changes in yield-related traits determined for the four cultivars under field **(A,C,E)** and glasshouse **(B,D,F)** conditions and under the two water regimes (control in blue and water deficit in gray): number of fruits per plant **(A,B)**, individual fruit fresh weight (FW) **(C,D)** and dry matter content of fruit pericarp collected at 55 DAA **(E,F)**. For **(A–D)**, mean ± SE, *n* ≥ 8. For **(E,F)**, mean ± SE, *n* ≥ 4. Bars marked by different letters indicate significant different values (Kruskal–Wallis test, α = 0.05).

Interestingly, under field-grown conditions, WD did not impact the fresh yield, expressed as total fruit biomass per plant, and yield-related traits (number of fruits and individual fruit fresh weight; Table 1), but it slightly increased the dry yield (up to +27% for “H1015”). We further analyzed the interactions between genotype, irrigation t and growing condition (Figures 2, 3). Overall in field under well-watered conditions, all cultivars reached almost similar fresh yields (on average 2,636 g plant^−1^ or 87 t ha^−1^), but different dry yields (Figures 2A,C, blue bars). The dry yield was the highest for “Terradou” end the lowest for “H1015” (+46% comparing “Terradou” to “H1015”). Under WD, “Terradou” reached the highest fresh and dry yields (Figures 2A,C, gray bars). The fruit dry matter content was higher under WD than under control condition, and the difference was significant for “H1015” and “Miceno” (+27% for “H1015” and +26% for “Miceno,” Figure 3E).

Under glasshouse conditions, the fresh and dry yields were similar for all cultivars, except “Terradou” which outperformed under WD (Figures 2B,D). In the glasshouse, WD significantly decreased the fresh and dry yields of all cultivars (from −34 to −44% for fresh yield and from −25 to −47% for dry yield Figures 2,B,D). This reduction resulted from a decrease in both the number of fruits per plant and the individual fruit fresh weight (Figures 3B,D). Under WD, the dry matter content of fruit pericarp did not significantly change in glasshouse (Figure 3F).

Comparing field and glasshouse experiments, fresh and dry yields of control plants were significantly higher in the glasshouse whatever the cultivar (up to +50% of fresh yield; Figures 2A,B). Overall, the mean fresh mass of individual fruit was lower in the glasshouse than in the field, but the number of fruits per plant was higher, especially under well-watered conditions (−22%, and +101%, respectively) (Figures 3A,C). On the other hand the fruit dry matter content was higher in the field than in the glasshouse, especially under WD (+22%; Figures 3E,F). The genotype effect was higher in field than in glasshouse (between 38 and 43% of the variance associated to genotype in field, Table 1).

In field, the low impact of WD on yield prompted us to calculate water use efficiency as the ratio between total fresh yield and total water used for irrigation (kg m^−3^). In field, the water use efficiency ranged from 25 to 29 kg m^−3^ (depending on cultivars) for control plants and from 33 to 41 kg m^−3^ for WD plants. “Terradou” presented the highest values under both water treatments. In the glasshouse, the water use efficiency ranged from 32 to 39 and from 38 to 50 kg m^−3^ for control and WD plants, respectively. The highest value was reached by “H1015” under control, whereas “Terradou” showed the highest efficiency under WD.

Table 2 synthetizes the effects of WD, genotype and maturity stage on quality traits analyzed by univariate ANOVAs. Fruit composition in soluble sugars, organic acids, and carotenoids was determined on a dry weight basis. None of the tests involving WD (including interactions) was significant except for Lycopene and citric acid under glasshouse conditions (Table 2). Fruit composition was mainly controlled by the genotype (between 26 and 57% of the variance associated to genotype in field and between 23 and 64% in glasshouse).

### Genotype and maturity stage controlled puree quality while WD improved rheological properties

**Figure 4:**
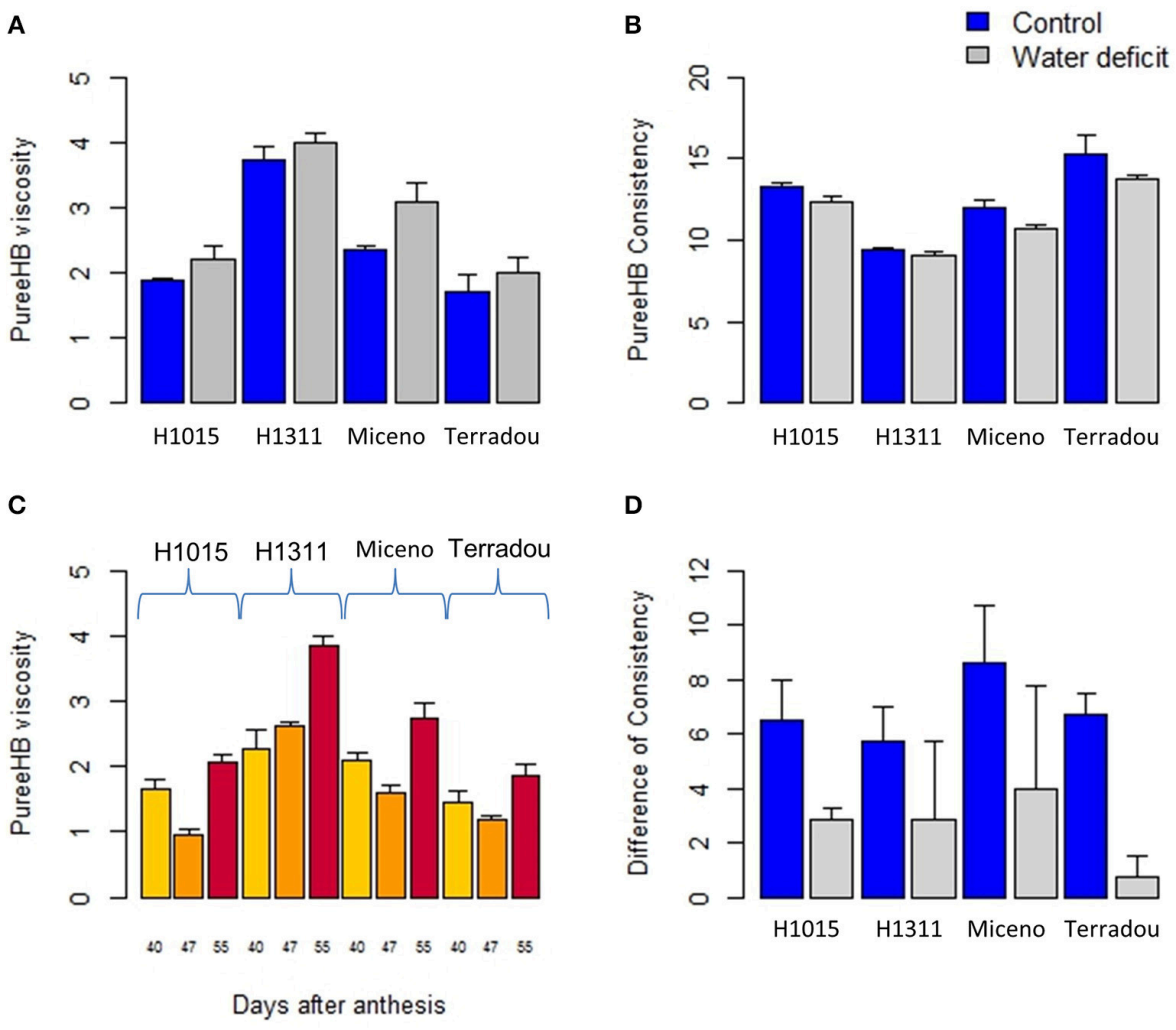
Impact of water deficit **(A,B,D)**, genotype **(A–D)** and maturity stage **(C)** on the rheology of puree, under field-grown conditions. **(A,B)** Changes in Hot break puree viscosity and Bostwick value, all fruits were collected at 55 DAA. **(C)** Variations in Hot break puree viscosity obtained with fruit when fruits were collected [40, 47, and 55 days after anthesis] **(C)**. **(D)** Difference of consistency (measured by Bostwick device) between Cold break and Hold break purees obtained from fruits collected at 55 DAA. Values are mean ± SE, *n* ≥ 2.

Tomatoes from field and glasshouse experiment were processed through CB and HB methods and puree quality was assessed based on viscosity and color parameters. In all tests, genotype and maturity stage effects were significant. The genotype effect was higher than the maturity stage effect, except for the Hue angle and the viscosity of CB purees from field (Table 2). A highly significant genotype × stage interaction was found for the viscosity of HB purees (Table 2). We further analyzed the interactions between genotype, maturity stage and irrigation treatment in field, and their effects on puree rheological properties (Figure 4). Cultivar “H1311” produced the most viscous purees in all situations (Figure 4). With regard to the maturity stage impact, viscosity slightly declined or was stable when fruits were harvested between 40 and 47 DAA and then sharply increased for fruits harvested between 47 and 55 DAA (up to +100%, Figure 4C). Interestingly, WD led to significant higher puree viscosity and consistency (Figures 4A,B). Within the WD group, the viscosity of “H1311” purees was 22.3, 44.36, and 49.62% higher than the viscosity of purees from “Miceno,” “H1015,” and “Terradou,” respectively. The ranking of genotypes was similar in the control group and consistent patterns of viscosity and Bw consistency were obtained. Another major finding of our study was that WD significantly influenced the fruit reactivity, assessed through the difference in consistency between HB and CB purees. For all genotypes, the reactivity was remarkably lower for purees produced from WD tomatoes than for purees from control tomatoes (Figure 4D). At 55 DAA, the losses in consistency ranged from −48% for “H1311” to −75% for “Terradou.”

The responses of puree viscosity to genotype, maturity stage and WD treatment were similar when fruits were produced under glasshouse conditions, but they differed in absolute values. Interestingly, purees from the glasshouse production were always less viscous when compared to the field production whatever the cultivar, the water regime or the maturity stage. The puree made from WD fruits produced in open-field showed 69.45% increase in viscosity and 21.67% decrease in Bw, while purees made from control fruits showed 68.46% increase in viscosity and 23.40% decrease in Bw, when compared to the glasshouse.

**Figure 5:**
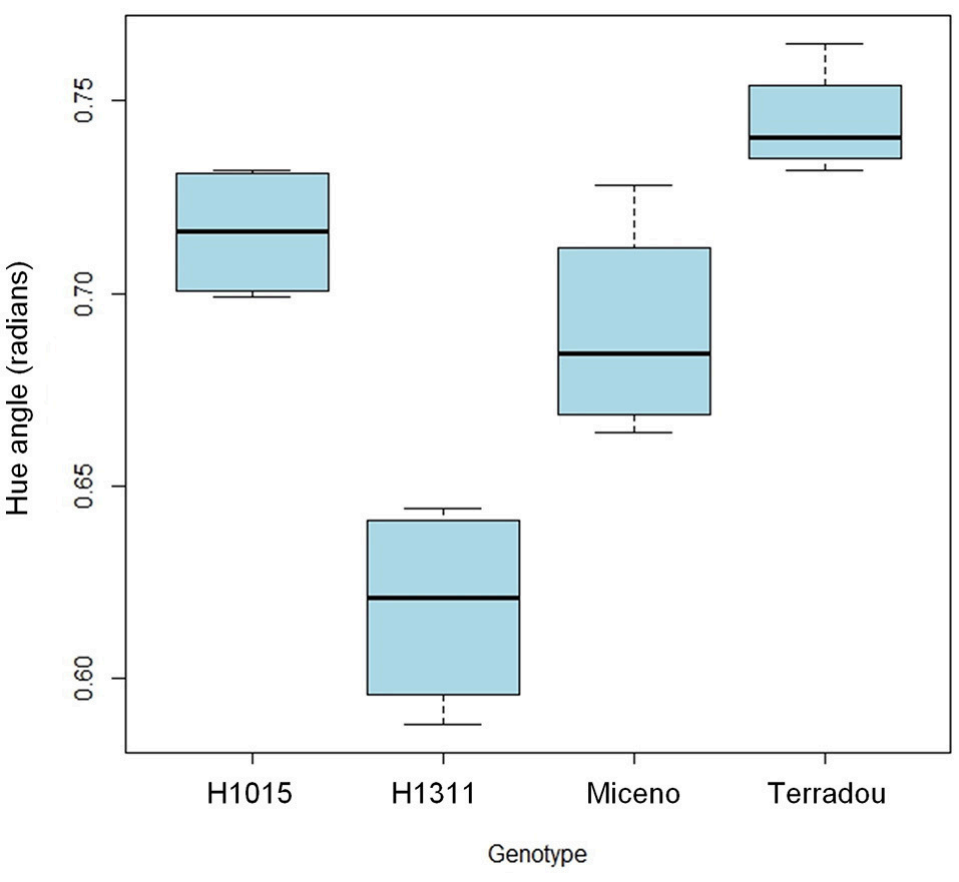
Impact of genotype on Hue angle calculated from color coordinates measured on Hot break puree obtained from fruits collected at 55 DAA and grown in open-field. Boxplots gather the values from fruits grown under the two water regimes since only genotype had a significant influence on color changes.

Regarding the color parameters under field-grown conditions, Hue angle values were significantly affected by genotype and maturity stage only. The hot break purees from “H1311” ripe fruits presented the lowest values (Figure 5), which was consistent with the lowest Hue angle of fresh fruits among cultivars. As expected, Hue angle values significantly varied according to the maturity stage.

### Correlation between fresh fruit and puree quality traits

**Figure 6:**
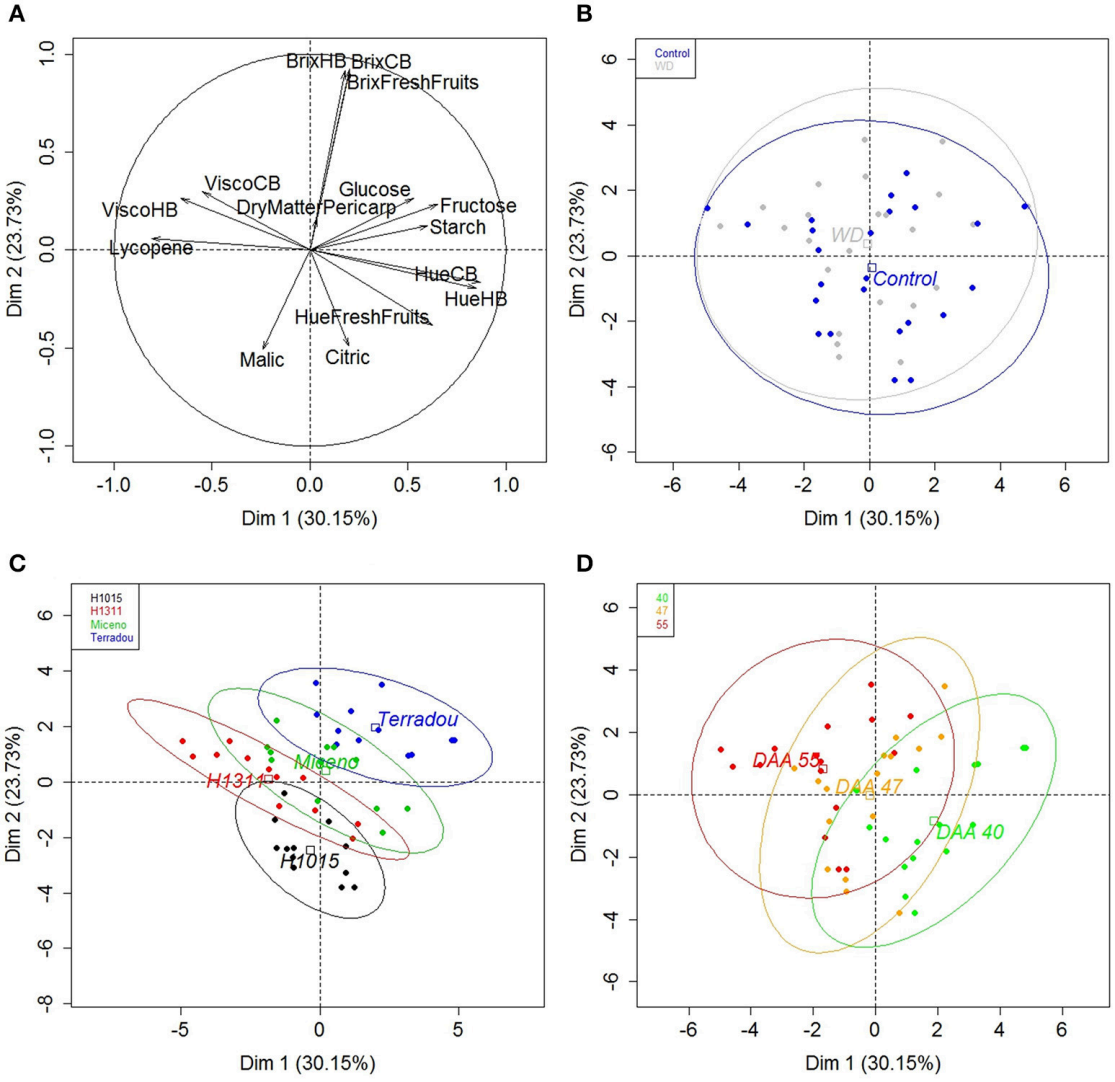
PCA results of fruit quality traits (°Brix of fresh fruits, dry matter content of pericarp, Hue angle of fresh fruits, starch, glucose, fructose, citric and malic acids, and lycopene determined on dry weight basis) and puree quality traits (°Brix, Hue angle, and viscosity of purees obtained after CB and HB processes) in field according to dimension 1 and 2 (53.88% of the total variance). The proportions of explained variability are indicated for each axis. **(A)** Projection of the quality traits taken into account; **(B–D)** Projection of individuals. Each point corresponds to the mean of three replicates. **(B)** Centers of gravity for water treatments, **(C)** Centers of gravity for genotypes, and **(D)** Centers of gravity for maturity stages.

**Figure 7:**
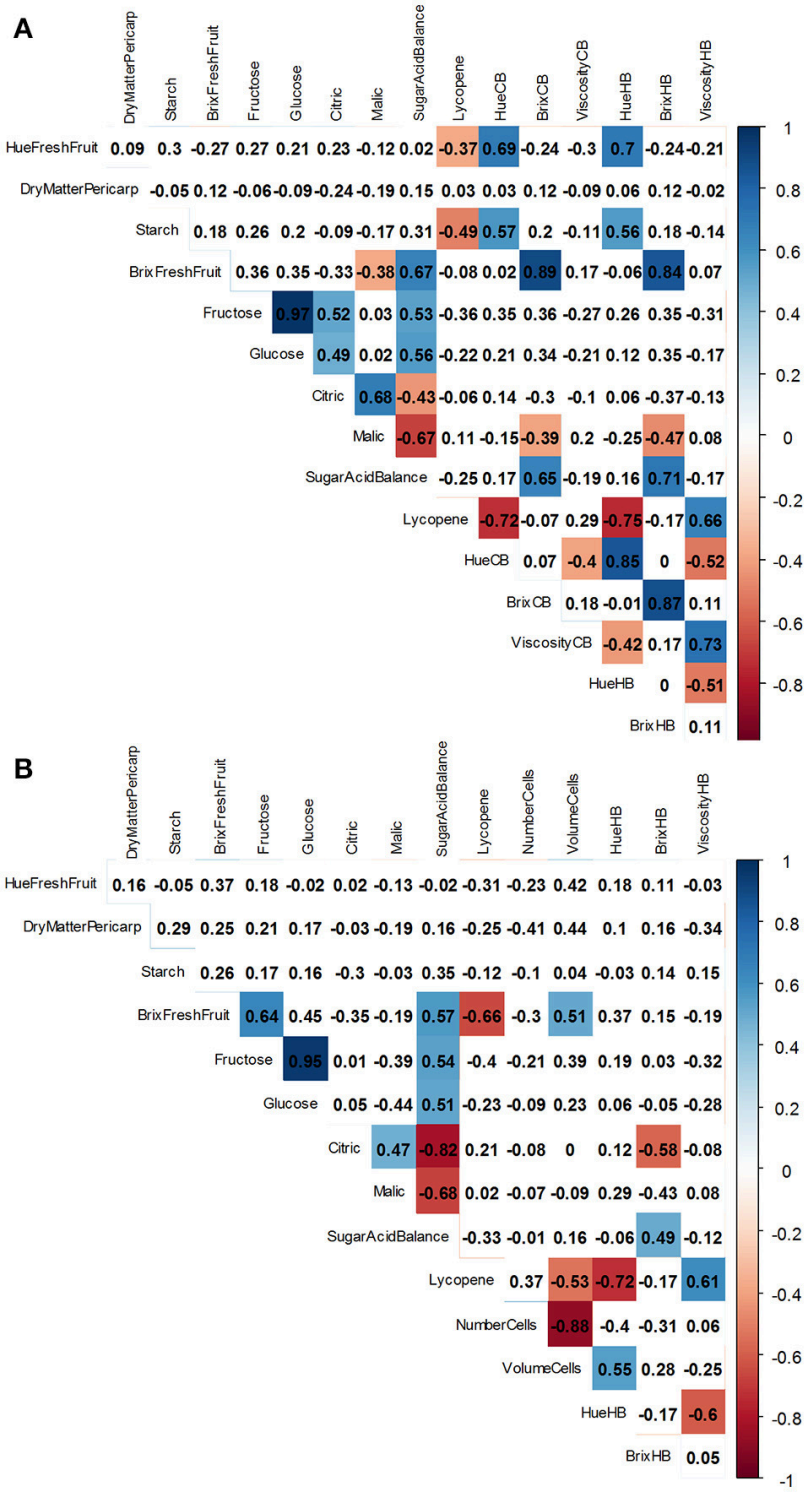
Correlation matrix of quality traits measured on fruit [Hue angle of fresh fruit, dry matter content of the pericarp, Brix, starch, fructose, glucose, citric, malic, and lycopene contents expressed on the dry weight basis, number and volume of cells of the pericarp] and on puree [Hue angle from cold break (CB) or hot break (HB) process, Brix CB or HB, and viscosity CB or HB] under field **(A)** and glasshouse **(B)** conditions. A color was associated to Pearson correlation coefficients from red (negative correlation) to blue (positive correlation) when correlations were significant (*P* < 0.001).

To understand the links between crop and process management, a PCA analysis was performed on fruit and puree quality traits under field conditions (Figure 6 and Supplementary Table 1), and under glasshouse conditions (Supplementary Figure 5 and Supplementary Table 2). The first and the second dimensions (Dim.) explained 54% of the total variance (Figure 6). The quality traits are plotted on the first two dimensions in (Figure 6A) while (Figures 6B–D) shows the projection of the observations and the centers of gravity for water treatments, genotypes, and maturity stages, respectively. Dim. 1 positively correlated with Hue HB, Hue CB, Hue fresh fruits, fructose, starch, and glucose and negatively correlated with lycopene, viscosity HB and viscosity CB (Figure 6A, and Supplementary Table 1). Fewer traits were well represented on Dim. 2: Brix HB, Brix CB, and Brix fresh fruits correlated with the positive values of Dim. 2 (Figure 6A, and Supplementary Table 1). The dry matter content of the pericarp was poorly represented on Dim. 1, 2, and 3. Citric and malic acids were poorly represented on Dim. 1 and 2, but contributed to Dim. 3 (Figure 6A, and Supplementary Table 1). The projection of individuals (Figures 6B–D) confirmed the effects of genotypes and maturity stages on fruit and puree quality (Figure 6). Cultivars were separated on Dim. 1 and 2 whereas Dim. 1 explained differences among maturity stages. As mentioned, some traits were poorly represented on the first two dimensions. Consequently, Pearson correlation correlations between fresh fruit and puree quality traits were analyzed further. Figure 7 summarizes data obtained under field (Figure 7A) and glasshouse (Figure 7B) conditions. Significant correlations are indicated by a color code: red for negative significant correlation and blue for positive significant correlation. Of the 120 pairs of traits, there were 32 significant correlations (*P* < 0.01) under field conditions (vs. 18 significant correlations among 105 pairs of traits under glasshouse conditions). As expected, color of CB and HB purees, determined by Hue angle values, positively and negatively correlated with, respectively, Hue values and lycopene contents of fresh fruits (Figure 7A). Brix of CB and HB purees positively correlated with Brix of fresh fruits. Some correlations were also found among fruit traits, such as individual sugars, individual acids, and sugar-acid balance or among puree traits such as CB and HB viscosities. Surprisingly, no correlation was found between puree viscosity and fruit dry matter content, starch, or Brix. It was also interesting to note that viscosity of HB purees positively correlated with lycopene contents of fresh fruits. There was a concordance between field and glasshouse experiments. However, the positive correlations between Hue angle of HB purees and Hue angle of fresh fruits or between Brix of purees and Brix of fresh fruits were not significant under glasshouse conditions (Figure 7B). On the other hand, a positive correlation was observed between the average pericarp cell size and the Hue angle of the puree.

**Figure 8:**
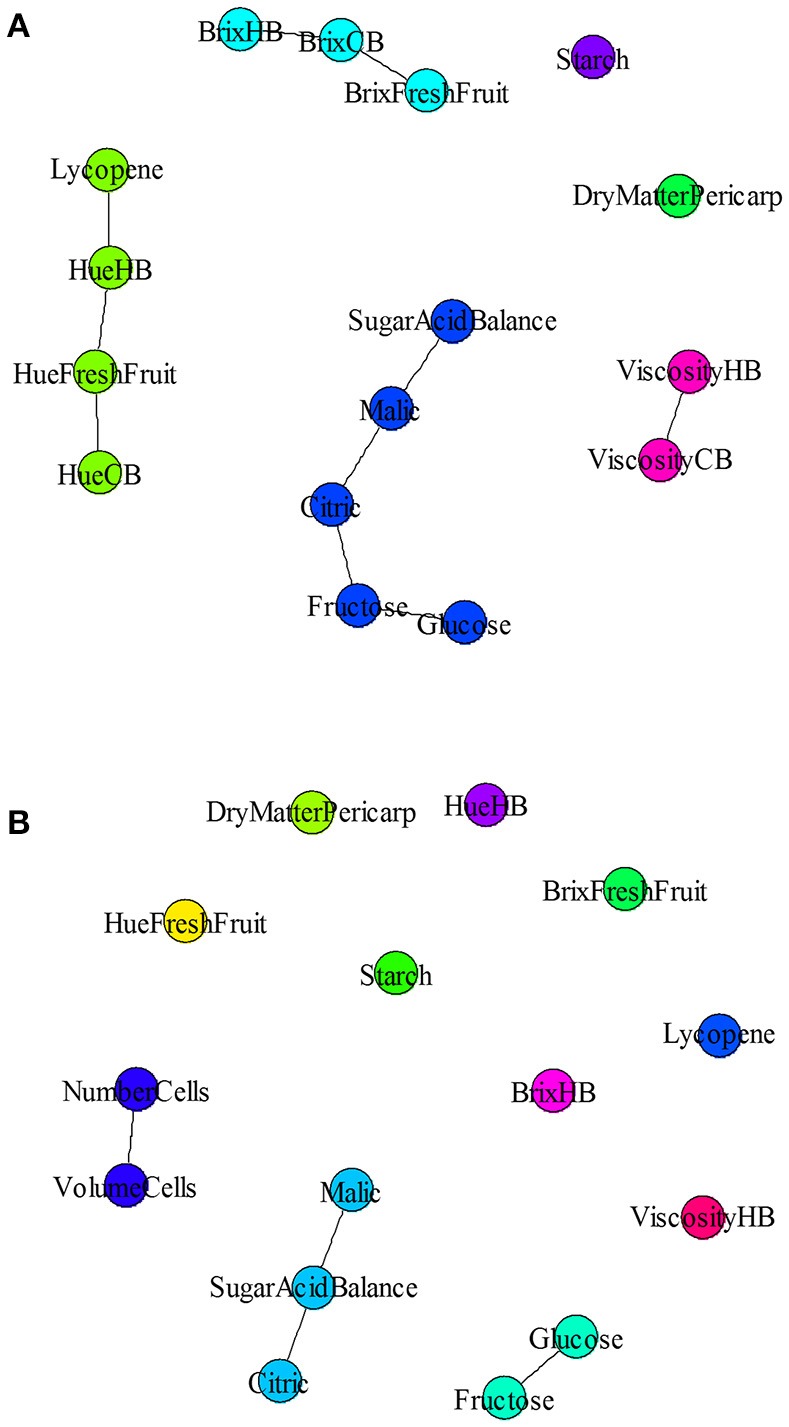
Partial correlation network for fruit quality and puree quality under field **(A)** or glasshouse **(B)** conditions. Lines indicate correlations between variables (*P* < 0.001).

A partial correlation network was built on fruit and puree quality traits, based on the residues of the linear regression (elimination of the genotype and treatment effects) to determine traits that were functionally related (Figures 8A,B). Partial correlation analysis was performed independently for the field (Figure 8A) and the glasshouse (Figure 8B) experiments. Correlations among fruit and puree quality traits were confirmed for color and Brix (Figure 8A). However, puree viscosity was not related to any trait of fruit quality (Figure 8A). Under glasshouse conditions, puree quality was unrelated to fresh fruit traits (Figure 8B).

## Discussion

The quality of tomatoes intended for processing is currently addressed on delivery through color and Brix, whereas other traits are overlooked. In the field, irrigation is currently stopped 1 week before harvest in order to increase the fruit dry matter content. During processing, part of the water is removed and the matrix is submitted to thermal treatments in order to control more or less empirically the final puree viscosity. Yet very few works provided an integrated view of the management of tomato puree quality from field to can. For this purpose insights into interactions between factors that drive fruit quality during the growing season and those that operate during processing should be gained. In this work we investigated fruit quality in response to water supply, genotypes and ripening stages, and we assessed their impact on puree quality obtained from HB and CB processes.

### Moderate effects of WD on fruit yield and quality are observed in glasshouse conditions only irrespective of the genotype

Water availability is one of the main factor impacting plant growth and consequently harvestable yield (Boyer, 1982; Tardieu et al., 2011; Katerji et al., 2013; Ripoll et al., 2014). For all genotypes, under glasshouse conditions, the effect of WD, as reflected by changes in water status and stomatal conductance, resulted in reductions in total plant biomass, fruit setting, and fruit fresh mass. The reduction of plant growth observed under WD is likely to originate from a sink, hydromechanical limitation rather than a source, photosynthetic limitation since growth is generally more affected by drought than carbon assimilation (Muller et al., 2011). Nonetheless, carbon supply could also represent a significant growth limitation in the fruit, where carbon-rich osmotica are required for sustaining expansive growth (Pantin et al., 2013). In addition, impairment of carbon supply may have been determinant for fruit set (D'Aoust et al., 1999). Under field conditions, WD reduced leaf area and total plant biomass, did not impact the total fresh yield, but slightly increased the total dry yield, and in average, increased the water use efficiency by 20% compared to control condition. The average total fresh yield obtained in this study corresponded to total yields commonly observed in France under well-watered conditions (80 t ha^−1^, http://www.sonito.fr). Total fresh and dry yields were genotype-dependent. These results are in agreement with previous works on the effects of moderate deficit irrigation on processing tomato. According to Patanè et al. (2011) a deficit irrigation at 50% ETc from flowering does not significantly reduce the total or marketable yields, but increases water use efficiency by about 40%. Similarly, Stikic et al. (2003) have shown that partial root drying (PRD) induces a significant reduction of total plant biomass without affecting fruit diameter and fresh mass. Accordingly, water use efficiency at crop level is increased by PRD treatment (Stikic et al., 2003).

Under glasshouse conditions, the effect of WD on yield was higher than the effect measured in field. In addition, substantially higher yields were achieved in glasshouse than in field. It may result from lower plant density and temperatures in glasshouse (Poorter et al., 2016). In glasshouse, inter-cultivar variations in total yields were observed under WD only. This suggests that the ranking of genotypes resulted from differential adaptation to WD in glasshouse and to abiotic factors other than WD in field (density or temperature).

In contrast to the negative effect on plant growth and fruit fresh mass, moderate water deficit has been reported to improve fruit quality of tomato (Pernice et al., 2010; Patanè et al., 2011; Barbagallo et al., 2013; Ripoll et al., 2016). The increase in fruit dry matter content in response to WD is well-known (Ripoll et al., 2014). However, whatever the genotype, we did not observe any change in sugar, acid, and lycopene contents, expressed on a dry weight basis, in response to WD. Ripoll et al. (2016) have already underlined that beneficial effects of moderate WD on fruit sugar, acid and carotenoid contents, reported on a fresh weight basis, mainly results from a dehydration effect, which is confirmed by our study. In addition, effects of WD on fruit dry matter composition strongly depend on genotype and stress intensity (Ripoll et al., 2016). In our study, the ranking of genotype in terms of fruit composition was not modified by the WD applied from flowering to harvest.

### Water deficit improves puree rheological properties

Consistency constitutes one of the main quality traits of tomato purees, which are considered as suspensions of insoluble particles (pulp) into an aqueous solution (serum) (Moelants et al., 2014). In tomato, fruit dry matter encompasses soluble (mainly sugars and acids) and insoluble (such as pectins and other polysaccharides) solids (Foolad, 2007). Insoluble solids are thought to determine puree viscosity (Davies et al., 1981). However, the precise role of the physicochemical properties of these solids remains unrevealed. Pectin composition and degradation were stressed out as major parameters, while some authors put solid particle size and shape on first stage (Sanchez et al., 2002; Lin H. et al., 2005; Moelants et al., 2014). All those parameters are regulated during fruit maturation and genetic control (Sanchez et al., 2002; Foolad, 2007; Anthon et al., 2011). Our results are in agreement since cultivar and maturity stage had major impacts on puree viscosity (Figure 5). Interestingly, the WD applied in our experiment improved puree viscosity of all cultivars, despite no change in fruit composition (on a dry weight basis) and no correlation between fruit dry matter content and puree viscosity. We propose that the effect of WD on puree rheology was driven by changes in pectin composition, and by changes in particle size and shape. Indeed, transcriptome analysis have revealed that plant response to drought includes differential cell wall synthesis and remodeling (Tenhaken, 2014). In addition, we found that WD decreased the loss of viscosity between HB and CB purees which suggests that the activity of pectin-degrading enzymes in fruits, produced under WD, was modified. The difference in rheology after HB and CB treatments is attributed to temperature effects on endogenous pectinolithic enzymes, namely polygalacturonase (PG) and pectin methyl-esterase (PME) involved in fruit softening (Anthon et al., 2002; Moelants et al., 2014). The involvement of these enzymes has been confirmed by HB/CB processing of genetically modified tomatoes (Errington et al., 1998), but the exact relationship between PG, PME and rheology remained partially obscure. Indeed CB treatment leads to different biochemical and physical properties of water soluble pectins when compared to HB (Lin H. et al., 2005; Lin H. J. et al., 2005). The proportion of water insoluble solids is not significantly different between CB and HB purees (Sanchez et al., 2002), but particle size and shape are also impacted by the breaking temperature (Errington et al., 1998). It has been shown in other plant species, that pectin-degrading enzymes can be down-regulated by water stress (Le Gall et al., 2015). Thus, the activity of pectin-degrading enzymes in response to WD should be analyzed in further details to disentangle the effect of pectinolithic enzymes from the effect of particles.

### Fruit lycopene content rather than dry matter content controls puree quality

Lycopene is the main pigment of red tomato cultivars (Fraser et al., 1994). It is well-established that color parameters measured with a chromameter provide a robust evaluation of lycopene contents determined by HPLC (Arias et al., 2000). So the strong correlations between fruit lycopene, fruit color parameters, and puree color parameters were not surprising. On the contrary the correlation between lycopene content of fresh fruits and puree viscosity is new.

Several studies reported relations between puree viscosity and fruit dry matter or soluble solid contents (Davies et al., 1981). However, such correlation was not observed in our study. Moelants et al. (2014) have reviewed relationships between food structure and rheological properties of plant-tissue-based food suspensions. Besides the importance of particle concentration already mentioned, particle size and particle morphology also appear to be key structural parameters controlling the rheological properties (Moelants et al., 2014), and especially, particle size distribution (Leverrier et al., 2016). Lycopene is known to be included in membrane-shaped structures of chromoplats (Egea et al., 2010). The correlation between lycopene content of fresh fruit and the puree viscosity prompts us to suggest that lycopene content improves puree rheological properties by enhancing the proportion of small particles of lycopene.

Our study demonstrated that challenging tomato producers to reduce water withdrawal could be eased by a better integration of the manufacturing requirements. Previous studies indicated that tomato fruit growth and quality were weakly impacted by moderate WD in glasshouse production, and our study confirmed that this result could be transposed in field production. A reduction of water supply from 100 to 60% of the ETP, increased the water use efficiency by 20% on average and may enhanced the dry yield depending on genotype. This result holds out some progress margin for the industry that spends most of its energy in water removal from raw material. We also found that WD modified the reactivity of tomato fruits to process. As a whole, the strong impact of genotype on puree's viscosity and the lack of correlation between puree viscosity and fruit soluble solid content or dry matter content, open interesting perspectives to better understand the links between crop and process management. Yield or fruit dry matter content matter a lot, but yet the condition of fruit production and their ability to produce high quality and stable industrial products should be considered as well.

## Author contributions

DP, NB, and AF: Planned and designed the research. AA, DP, RG, and AF: Performed experiments. AA, DP, NB, and AF: Analyzed the data and wrote the manuscript.

### Conflict of interest statement

The authors declare that the research was conducted in the absence of any commercial or financial relationships that could be construed as a potential conflict of interest.

## Abbreviations

Bw: Bostwick
ETP: evapotranspiration
PG: polygalacturonase
PME: pectin methyl-esterase
SSC: soluble solid content
WD: water deficit.
