# Supplementary material for: Combined Effects of Irrigation Regime, Genotype, and Harvest Stage Determine Tomato Fruit Quality and Aptitude for Processing into Puree
Source: Front Plant Sci. 2017 Oct 5;8:1725. doi: 10.3389/fpls.2017.01725 (PMC5633859; doi:10.3389/fpls.2017.01725)
Supplement: Supplementary file 1 [file Presentation1.pdf]

Supplementary Material

Combined effects of irrigation regime, genotype, and harvest stage determine tomato fruit quality and aptitude for processing into puree

Alexandre Arbex de Castro Vilas Boas, David Page, Robert Giovinazzo, Nadia Bertin, Anne-Laure Fanciullino*

*** Correspondence:** Anne-Laure Fanciullino: anne-laure.fanciullino@inra.fr

1 Supplementary Figures and Tables

1.1 Supplementary Figures

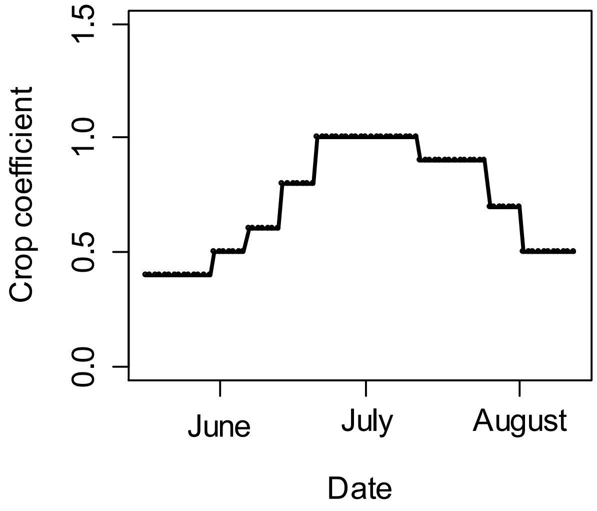

Supplementary Figure 1. Variations of tomato crop coefficient (Kc) during the 2016 season, from June to August.

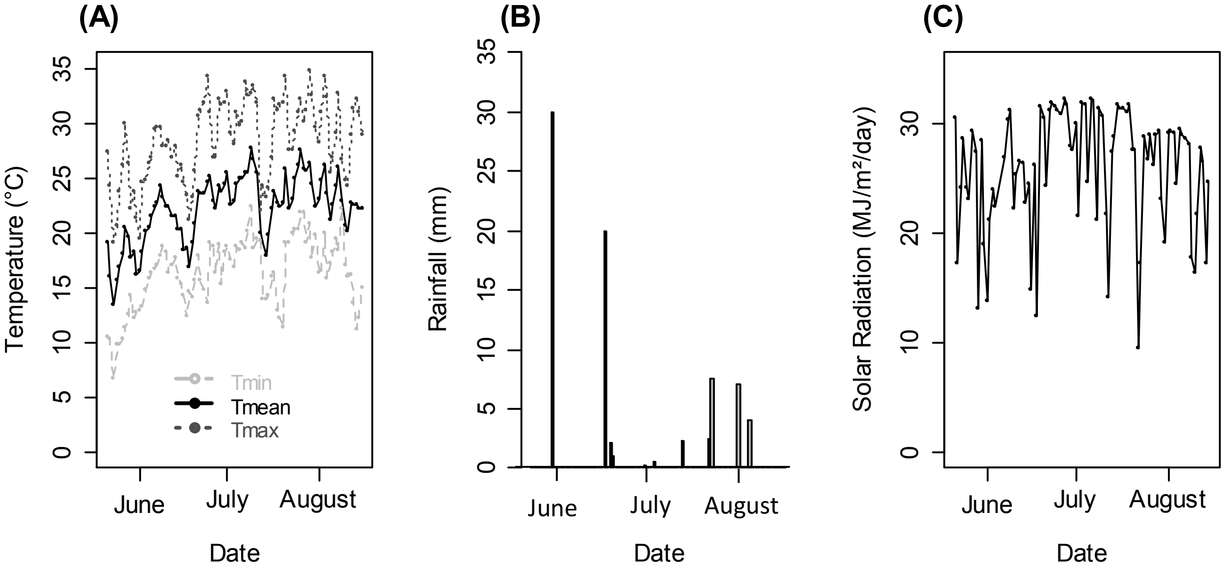

Supplementary Figure 2. A, Daily minimum (Tmin), mean (Tmean), and maximum (Tmax) air temperatures monitored in the open-field. B, Rainfall recorded daily during the time-course of the open-field experiment. C, Daily solar radiation recorded throughout the experiment.

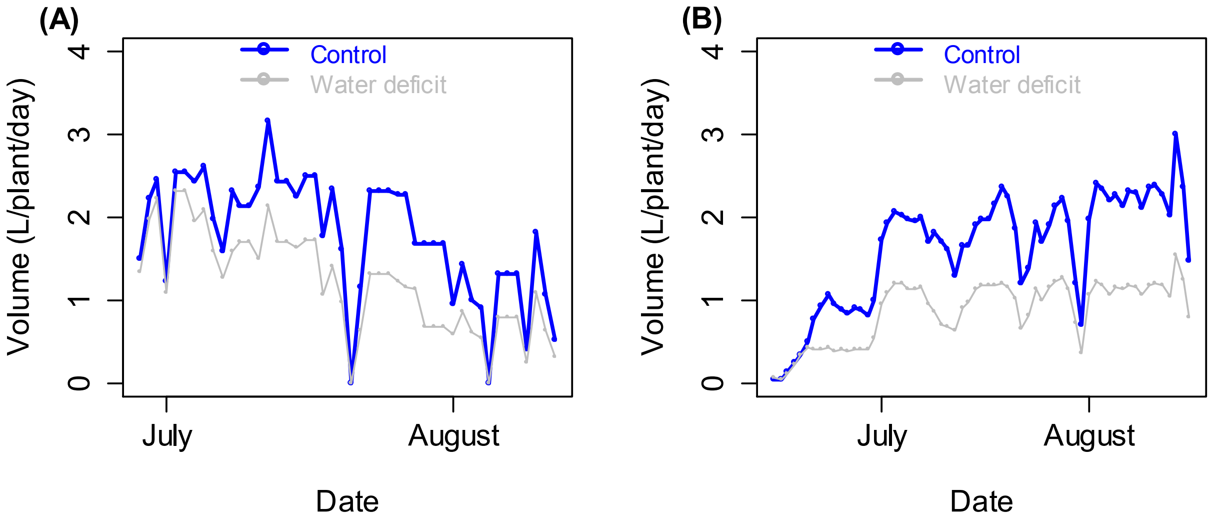

Supplementary Figure 3. Representation of the two irrigation regimes from June to August 2016 in A, the open-field and B, in the glasshouse. The control plants were irrigated in order to meet the evapotranspiration demand (ETP), which corresponded to a volume ranged from 0 to 3 L per plant and per day, throughout the experiments (blue lines). The irrigation was reduced to 60% (field) and 50% (glasshouse) of the ETP for the plants under water deficit (grey line).

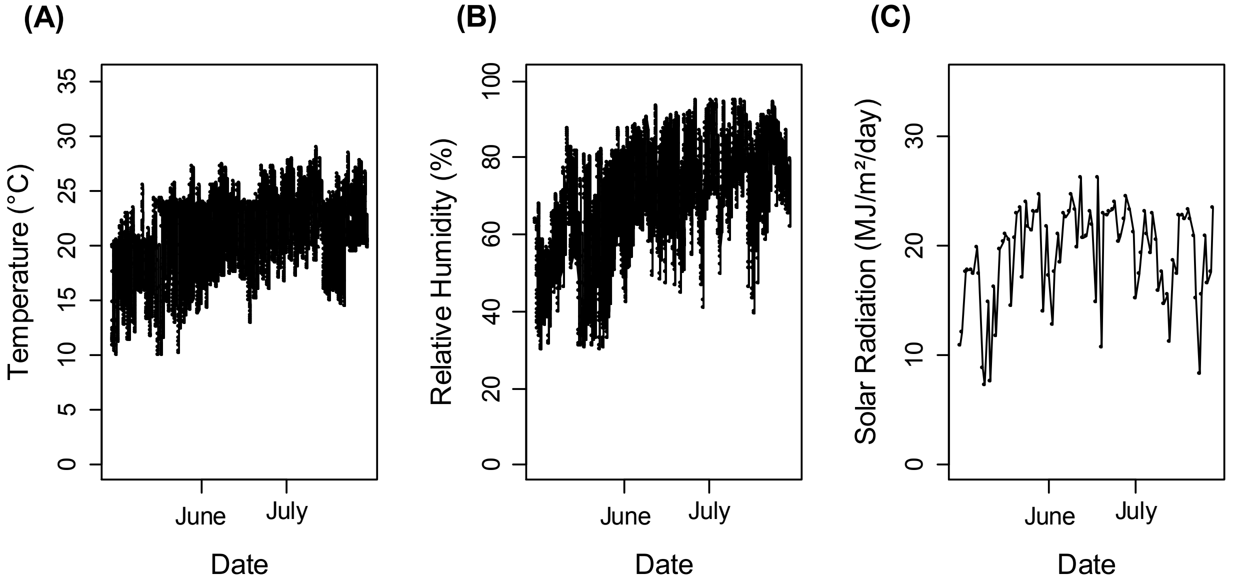

Supplementary Figure 4. Variations in temperature (A), and relative humidity (B) monitored every 30 minutes, and solar radiation (C) inside the glasshouse throughout the experiment.

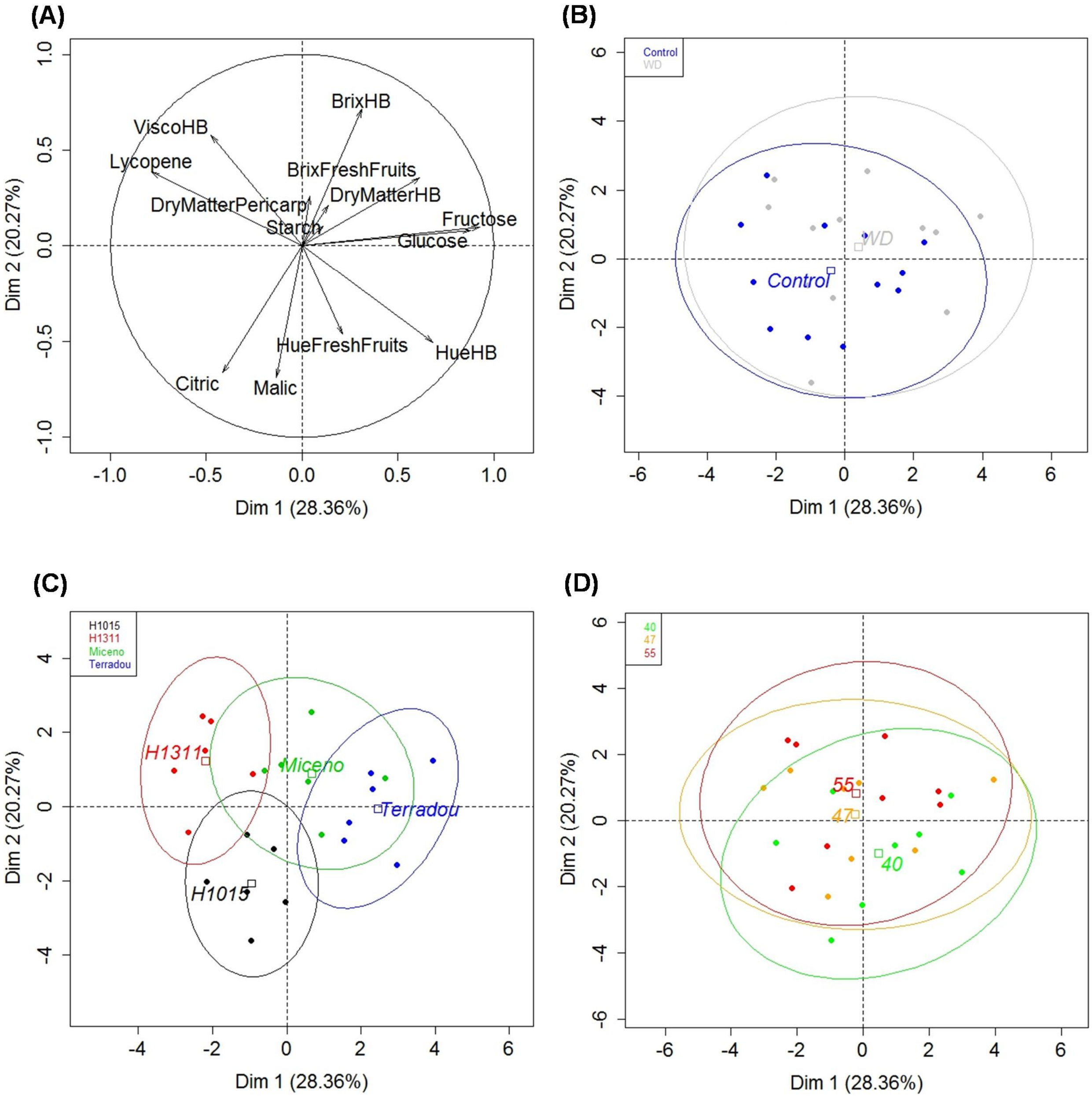

Supplementary Figure 5. PCA results of fruit quality traits (determined on dry weight basis) and puree quality traits in glasshouse according to dimension 1 and 2 (48.63% of the total variance). The proportions of explained variability are indicated for each axis; A: projection of the variables taken into account; B, C, and D : projection of individuals. Each point corresponds to the mean of four replicates. B, Centres of gravity for water treatments, C, Centres of gravity for genotypes and D, Centres of gravity for maturity stages.

1.2 Supplementary Tables

Supplementary Table 1. Outputs of PCA on fruit and puree quality traits in field

Eigenvalues	Dim.1	Dim.2	Dim.3	Dim.4	Dim.5	Dim.6	Dim.7	Dim.8	Dim.9	Dim.10	Dim.11	Dim.12	Dim.13	Dim.14	Dim.15
Variance	4.523	3.559	2.298	1.285	1.017	0.779	0.526	0.352	0.189	0.142	0.114	0.085	0.071	0.053	0.007
% of var	30.154	23.729	15.321	8.565	6.780	5.193	3.503	2.349	1.257	0.944	0.760	0.566	0.476	0.355	0.048
Cumulative % of Var	30.154	53.883	69.203	77.768	84.548	89.741	93.245	95.594	96.851	97.796	98.555	99.121	99.597	99.952	100.000

Traits (the 10 First)	Dim.1	ctr	cos2	Dim.2	ctr	cos2	Dim.3	ctr	cos2
HueFreshFruits	0.626	8.655	0.391	-0.384	4.139	0.147	-0.123	0.655	0.015
DryMatterPericarp	0.035	0.028	0.001	0.163	0.747	0.027	-0.317	4.386	0.101
BrixFreshFruits	0.178	0.704	0.032	0.914	23.479	0.836	0.089	0.346	0.008
Citric	0.196	0.853	0.039	-0.489	6.721	0.239	0.785	26.844	0.617
Malic	-0.241	1.284	0.058	-0.505	7.161	0.255	0.583	14.789	0.340
Glucose	0.528	6.167	0.279	0.265	1.977	0.070	0.728	23.077	0.530
Fructose	0.652	9.399	0.425	0.232	1.515	0.054	0.681	20.164	0.463
Starch	0.600	7.971	0.361	0.122	0.419	0.015	-0.176	1.341	0.031
Lycopene	-0.806	14.347	0.649	0.058	0.094	0.003	0.146	0.932	0.021
HueHB	0.848	15.895	0.719	-0.194	1.053	0.037	-0.320	4.455	0.102

Supplementary Table 2. Outputs of PCA on fruit and puree quality traits in glasshouse

Eigenvalues													
	Dim.1	Dim.2	Dim.3	Dim.4	Dim.5	Dim.6	Dim.7	Dim.8	Dim.9	Dim.10	Dim.11	Dim.12	Dim.13
Variance	3.687	2.635	2.162	1.095	0.837	0.642	0.551	0.480	0.401	0.285	0.129	0.086	0.011
% of var.	28.363	20.271	16.628	8.421	6.435	4.940	4.237	3.692	3.081	2.193	0.989	0.663	0.088
Cumulative	28.363	48.634	65.261	73.682	80.117	85.057	89.294	92.987	96.068	98.261	99.249	99.912	100.000

Traits	(the	10	first)						
	Dim.1	ctr	cos2	Dim.2	ctr	cos2	Dim.3	ctr	cos2
BrixFreshFruits	0.615	10.254	0.378	0.354	4.757	0.125	-0.169	1.325	0.029
BrixHB	0.313	2.657	0.098	0.712	19.245	0.507	-0.114	0.598	0.013
Citric	-0.413	4.623	0.170	-0.664	16.711	0.440	-0.108	0.537	0.012
DryMatterHB	0.136	0.504	0.019	0.212	1.702	0.045	0.770	27.445	0.593
DryMatterPericarp	0.042	0.049	0.002	0.259	2.544	0.067	0.555	14.247	0.308
Fructose	0.927	23.306	0.859	0.096	0.349	0.009	-0.062	0.175	0.004
Glucose	0.880	21.005	0.775	0.083	0.259	0.007	-0.118	0.640	0.014
HueFreshFruits	0.210	1.195	0.044	-0.463	8.148	0.215	0.695	22.341	0.483
HueHB	0.684	12.688	0.468	-0.505	9.679	0.255	0.208	2.009	0.043
Lycopene	-0.787	16.783	0.619	0.384	5.605	0.148	0.263	3.198	0.069
